# Modulation of behavioral responses and CA1 neuronal death by nitric oxide in the neonatal rat's hypoxia model

**DOI:** 10.1002/brb3.1841

**Published:** 2020-09-16

**Authors:** Zohreh Ghotbeddin, Zahra Basir, Javad Jamshidian, Farideh Delfi

**Affiliations:** ^1^ Department of Physiology Faculty of Veterinary Medicine Shahid Chamran University of Ahvaz Ahvaz Iran; ^2^ Stem Cell and Transgenic Technology Research Center Shahid Chamran University of Ahvaz Ahvaz Iran; ^3^ Department of Histology Faculty of Veterinary Medicine Shahid Chamran University of Ahvaz Ahvaz Iran; ^4^ Department of Pharmacology Faculty of Veterinary Medicine Shahid Chamran University of Ahvaz Ahvaz Iran

**Keywords:** behavioral performance, hippocampal histomorphometric changes, hypoxia, neonatal rat, nitric oxide

## Abstract

**Introduction:**

Neonatal hypoxia leads to cognitive and movement impairments that might persist throughout life. Hypoxia impairs hippocampal blood circulation and metabolism. The exact mechanisms underlying hypoxia‐induced memory impairment are not fully understood. Nitric oxide (NO) is a key neuromodulator that regulates cerebral blood flow. In this study, we aimed to evaluate the possible role of NO on behavioral and histomorphometric changes in the hippocampus following hypoxia in neonate rats.

**Material and methods:**

Neonate male rats (*n* = 28) were randomly divided into 4 groups: control, hypoxia, hypoxia plus L‐NAME (20 mg/kg), and hypoxia plus L‐arginine (200 mg/kg). Drugs were injected intraperitoneally for seven consecutive days. Hypoxia was induced by keeping rats in a hypoxic chamber (7% oxygen and 93% nitrogen intensity). Ten to 14 days after hypoxia, behavioral changes were measured using a shuttle box, a rotarod, and an open field test. The histological changes in the hippocampus were measured using H&E and Nissl staining methods.

**Results:**

Findings showed that hypoxia caused significant atrophy in the hippocampus. Furthermore, the administration of L‐NAME decreased the atrophy of the hippocampus in comparison with the hypoxic group. Behavioral results showed that hypoxia impaired memory performance and motor activity responses. Additionally, the administration of L‐NAME improved behavioral performance in a significant manner compared with the hypoxic group.

**Conclusions:**

Hypoxia damaged the neurons of hippocampal CA1 region and induced memory impairment. The NOS inhibitor, L‐NAME, significantly attenuated the negative effects of hypoxia on behavior and observed changes in the hippocampus.

## Graphical abstract

Because there are many conflicting reports on the role of NO in learning and memory processes following brain injury, in this work, we induced hypoxia model in neonate rats as a sensitive period of synaptic plasticity formation on memory, and after exposing neonate rats to hypoxia, to evaluate the neuromodulatory effect of NO, we tested memory and histomorphometric changes in adult rat offspring in the presence of NO agonist and antagonist. Our main research findings were memory impairment and hippocampus cell atrophy in hypoxia rat, whereas the NOS inhibitor, L‐NAME, significantly attenuated the negative effects of hypoxia on behavior and observed changes in the hippocampus.

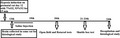

## 1 INTRODUCTION

Neonatal hypoxia is a burdensome critical healthcare problem worldwide which leads to the death of human infants or might result in lifelong sensory, motor, and cognitive impairments. Globally, 23% of neonatal death occurs due to the neonatal hypoxia (Odd, Heep, Luyt, & Draycott, 2017) Among the survivors, about 25% develop lifelong cognitive and sensory problems because of the severe damage to the brain. Insufficient blood supply and oxygen to the brain can lead to neonatal seizures, and many infants are born with acute respiratory failure and experience hypoxia (Shetty, 2015). The hippocampus region of the brain is highly susceptible to the functional and structural insult caused by hypoxia and acute respiratory distress syndrome (ARDS). Hypoxia results in bilateral pathological changes in the hippocampus at an early age and precipitates memory problems in childhood (Cooper et al., 2013). Oxygen deficiency during the critical period of synaptic maturation may cause cognitive impairment and autism‐like behaviors in the long term (Berg, 2011). Hypoxia can influence the synaptic plasticity and diminish long‐term potentiation (LTP) in the CA1 neurons of the hippocampus (Zhou, Bell, Sun, & Jensen, 2011). Since synaptic plasticity has particular importance in the formation of LTP and memory formation, synaptic plasticity dysfunction can cause long‐lasting learning and memory impairments. Hypoxia and ischemia create significant neuropathological changes and affect cognitive ability through circulation and brain metabolism disturbances (Gale & Hopkins, 2004). Yao et al. (2016) has suggested that the morphological and structural changes occur in neonatal rat brain after hypoxic‐ischemia which can be detected using Nissl staining.

Nitric oxide (NO), as a gaseous neurotransmitter, easily crosses the cell membrane. Animals studies have shown that NO is involved in numerous neuronal functions including learning and memory processes, pain, vasodilatation, and immune responses (Butler & Williams, 1993). In the neurons, NO is synthesized by nitric oxide synthase (NOS) from the conversion of L‐arginine into L‐citrulline. At physiological dosages, NOS plays a critical role as a modulator of synaptic plasticity, LTP formation, and long‐term memory consolidation (Prast & Philippu, 2001). In the hippocampus, the NMDA receptors initiates synthesis of NO which further strengthen the neuronal processes regulating the learning and memory (Garthwaite, 1991). Reports have demonstrated that the NOS activity in the rat brain increases up to 45% immediately after learning task (Bon & Garthwaite, 2003). NO can enhance the oxygen delivery to neurons through vasodilatation of brain vessels and nerve cell perfusion, facilitating learning and memory processes (Bolanos & Almeida, 1999). Nitric oxide is a key factor in the brain pathophysiological responses to hypoxia. At least three isoforms of NO synthase have been identified: neuronal (nNOS), endothelial (eNOS), and inducible (iNOS) (Knowles & Moncada, 1994). Cerebral ischemia initiates a series of events that occur through glutamate excitatory amino acids and activates calcium‐dependent NOS isoforms such as nNOS and eNOS. The ischemia also activates iNOS in the glial cells with some delay. The activation of nNOS and iNOS contributes toward the development of ischemic brain injury (Ikonomidou & Turski, 1996).

There are conflicting reports about the role of NO on neuronal activity after combined ischemia‐hypoxia. Some reports have mentioned a protective effect, while others have proposed a toxic effect for NO following hypoxia. Increased NO synthesis in pathological conditions can be neurotoxic and cause neurodegeneration. Energy depletion, lipid and protein peroxidation, protein nitrosylation, and DNA damage following the production of free radicals are mechanisms by which increased NO synthesis leads to neuronal death (Bolaños et al., 1997). According to in vitro studies, increased production of peroxynitrite anion (ONOO_3_) from NO radicals may be a potential mechanism for neurotoxicity (Beckman, Beckman, Chen, Marshall, & Freeman, 1990). Many in vivo studies also have confirmed an increase in NO synthesis after brain injury using electron paramagnetic resonance (EPR). The studies suggested that after ischemia, NO radical synthesis often increases in specific brain areas such as the cortex, hippocampus, hypothalamus, amygdala, and substantia nigra (Kuppusamy, Ohnishi, Numagami, Ohnishi, & Zweier, 1995). The iNOS activity in glial cells also increases following ischemia, and the increased synthesis of NO radicals in neuroglia and neurons became neurotoxic. Hence, the application of a specific inhibitor may have protective effects on neurons (Kawase et al., 1996).

Considering the conflicting reports on the role of NO in learning and memory processes following brain injury resulting from ischemia and hypoxia, the present study aimed to evaluate the neuromodulatory effect of NO following neonatal hypoxia, as a sensitive period of synaptic plasticity formation on memory and histomorphometric changes of hippocampus in the presence of NO agonist and antagonist in rats.

## 2 METHODS AND MATERIALS

### 2.1 Animals

In this study, 10‐ to 12‐day‐old Wistar rats were used. All procedures were designed and executed according to the Ethics Committee recommendations for laboratory animals at Shahid Chamran University of Ahvaz and were in compliance with the NIH guideline for the care and use of laboratory animals. The rats were maintained in a fully controlled standard animal facility (12:12‐hr light: dark cycle at 22 ± 2°C and 55 ± 5% humidity). All animals had ad libitum access to water and food.

### 2.2 Animal model of hypoxic brain injury and drug treatments

**FIGURE 1:**
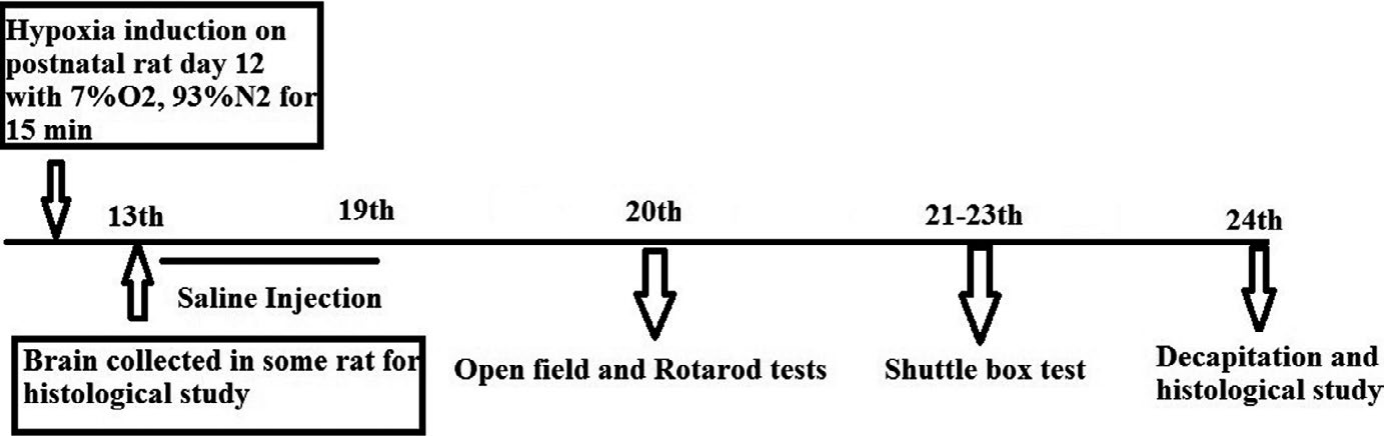
Schematic diagram of the experimental procedure in the hypoxia group

**FIGURE 2:**
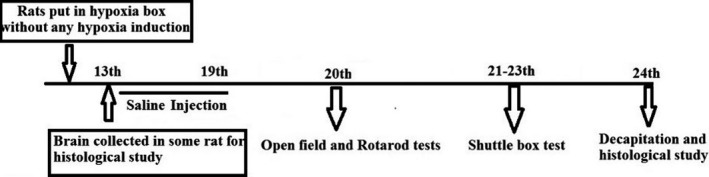
Schematic diagram of the experimental procedure in the sham group

**FIGURE 3:**
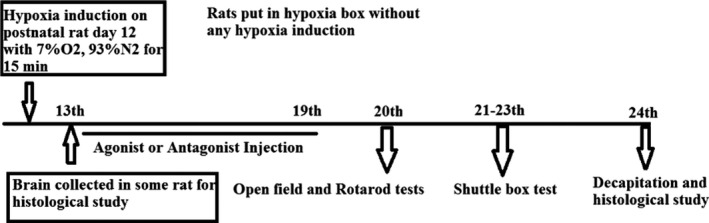
Schematic diagram of the experimental procedure in the L‐arginine and L‐NAME groups

Animals were randomized to the 2 major study groups: sham and hypoxia. Further the hypoxic group rats were treated with the L‐NAME (NOS inhibitor, 20 mg/kg) and L‐arginine (NOS precursor, 200 mg/kg) separately. The dose chosen were described earlier somewhere else (Kumura, Kosaka, Shiga, Yoshimine, & Hayakawa, 1994) (Hosseini et al., 2010). To induce hypoxia, 10‐ to 12‐day‐old rats were kept inside the airtight container similar to an incubator and were exposed to the 7% oxygen and 93% nitrogen for 15 min (Figure 1). As this model induces hypoxia during a sensitive period of synaptic plasticity, it can cause serious damages to neurons and long‐term motor and memory‐related disorders (Sedláčková et al., 2014). In sham group, rats were kept inside the hypoxia chamber for 15 min without inducing hypoxia, and immediately after the session, they received an intraperitoneal injection of normal saline (Figure 2), whereas the interventions, L‐NAME and L‐arginine groups, were injected intraperitoneally after the induction of hypoxia (Figure 3).

### 2.3 Behavioral tests

#### 2.3.1 Open field test

In the open field test, locomotor activity including frequency of rearing, movement velocity, and total distance travelled were monitored and analyzed using a video‐tracking system for 10 min on the postnatal week (P20) in a black plexiglass box (60 × 60 × 25 cm) (Bale et al., 2000).

#### 2.3.2 Rotarod test

The rotarod test was used to assess motor performance in the postnatal rats (P20). The animals were placed on the testing rod rotating at an initial speed of 5 rotations per minute (rpm). Then, the rod speed increased gradually to 45 rpm over 300 s. The time spent on the rod was recorded automatically for each animal. Each animal was initially given two opportunities for adaption to the device and then was tested three more times. The average time was calculated and considered for the analysis (Rustay, Wahlsten, & Crabbe, 2003).

#### 2.3.3 Passive avoidance test (Shuttle box)

The Shuttle box was composed of a dark and a light chamber, and the animal received shocks in the dark one. The test had three stages: training, testing, and acquisition (remembering) phase. Training phase: The animal was allowed to move freely into the light and dark chamber (without receiving any shock) for 5 min. Testing phase: Twenty‐four hours after training, the animals received electric shock (frequency of 50 Hz and intensity of 0.5 mA for 2 s) in the dark chamber. Acquisition phase: In order to test long‐term memory, 24 hr after training, the animal was put in the light chamber and the latency of entrance to the dark chamber and the time spent in the dark chamber were measured as indicators of passive avoidance memory (Sohrabi, Ghotbeddin, & Tabandeh, 2017).

### 2.4 Histomorphometric studies

#### 2.4.1 H&E Staining

Hematoxylin and eosin (H&E) staining was used to study the brain tissue morphology. For H&E staining, the animals were anesthetized and their brains were collected and preserved for 48 hr in 10% formalin buffer (Merck, Germany) solution at room temperature. Then, the brain tissues were dehydrated and embedded in paraffin. Serial sections (5–7 μm thickness) were taken and stained. The sections were dewaxed twice in xylene (5–15 min for each time) and dehydrated in serial ethanol (100%, 100%, 90%, 80%, and 70%, for 1 min, respectively). After rinsing by tap water for 3 min, the sections were stained with hematoxylin for 8 min and then rinsing again for 5 min followed by staining with eosin solution for 2 min. The samples were dehydrated, cleaned, and mounted. The cytomorphological changes of the brain tissues were observed by the light microscope (Olympus, Japan) at 200X magnification (Fischer, Jacobson, Rose, & Zeller, 2008).

#### 2.4.2 Nissl Staining

To assess neuronal cell loss and morphology of neurons in the hippocampal CA1 region, 6 rats were randomly selected from each group at two periods of time: (1) immediately after hypoxia (P13) and (2) at the end of behavioral tests (P24). Their brains were collected and fixed in 10% formalin buffer (Merck, Germany) solution. Ten μm coronal sections were obtained using a microtome. Each section was dewaxed, dehydrated, and stained with Cresyl violet acetate 0.1% for 5–10 min and then washed with distilled water twice (for several seconds each time). The slides were dehydrated in serial ethanol and xylene for 5 min twice and mounted. The cell density in the CA1 region was recorded using Dino‐Lite Digital Microscope. Neuronal cells were positively identified by their positive Nissl staining. The morphology of positively stained nuclei in the CA1 region was observed under a light microscope (Olympus, Japan) at 200× magnification (Kádár, Wittmann, Liposits, & Fekete, 2009).

### 2.5 Statistical analysis

The data are presented as the mean ± *SEM*. Normality was assessed using the Kolmogorov–Smirnov test. A one‐way ANOVA test followed by Tukey post hoc was used for comparing the means in different experimental groups. The level of significance was set at 0.05 for all the performed statistical tests. All statistical assessments were performed using the Statistical Package for Social Sciences (SPSS) software (version 21).

## 3 RESULTS

### 3.1 Passive avoidance test

**FIGURE 4:**
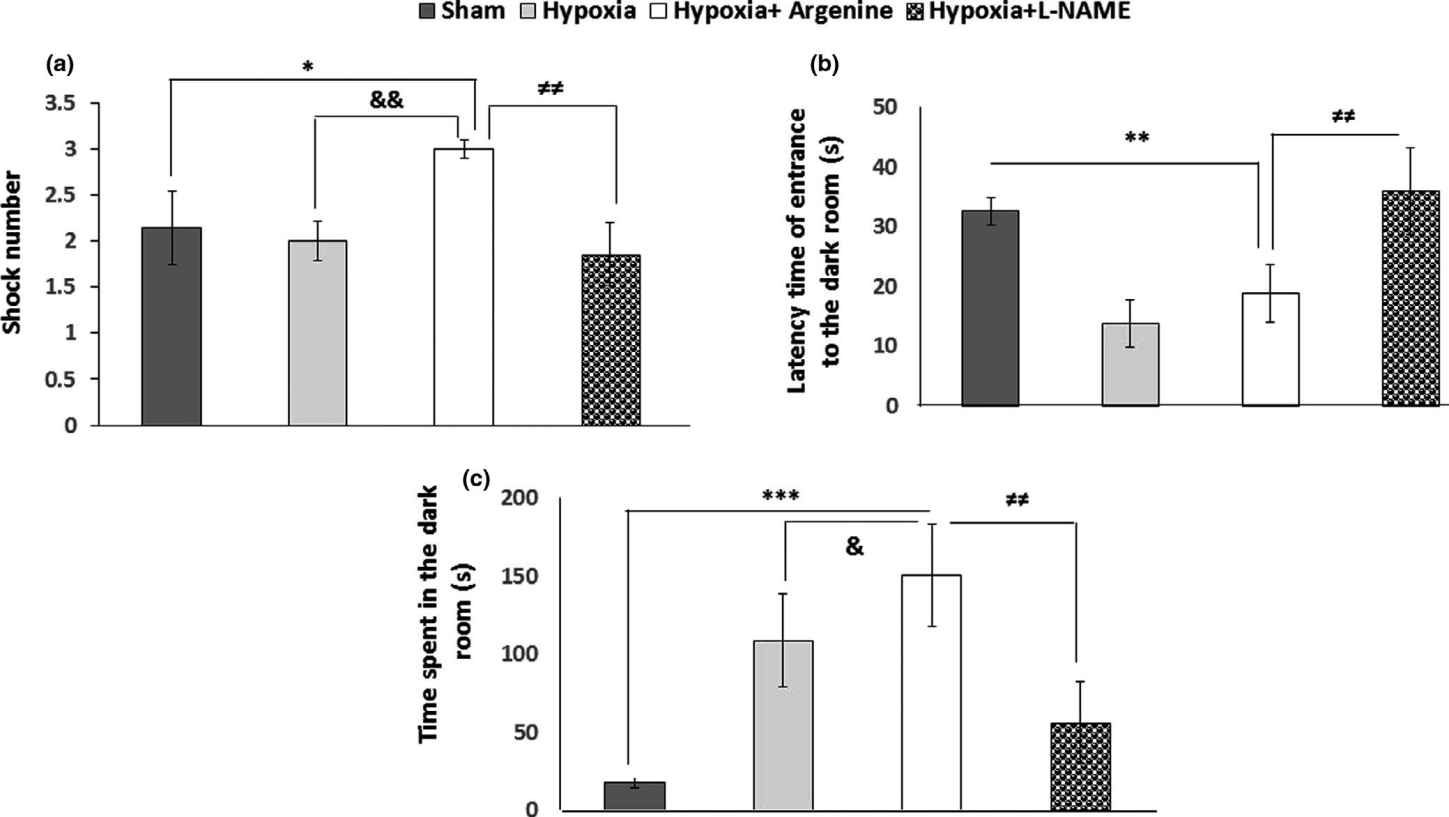
Effect of NOS agonist and antagonist on the learning behavior of hypoxic rats. (a) The number of shocks, (b) latency time of entrance to the dark room, and (c) time spent in the dark room were measured. Values represent the mean ± *SD*, *p < .05; **p < .01, and ***p < .001, L‐arginine‐treated rats versus sham rats; &*p* < .05; &&*p* < .01, L‐arginine versus hypoxia groups and ≠≠*p* < .01 L‐arginine‐treated rats versus L‐NAME‐treated rats

The results showed a significant increase in number of shocks in the L‐arginine group (3 ± 0.1) as compared to the sham (2.14 ± 0.40), hypoxia (2 ± 0.21), and L‐NAME‐treated groups (1.85 ± 0.34), respectively, *p* = .035, *p* = .015 and *p* = .006 (Figure 4a). In the L‐arginine‐treated group, the latency time of entrance to the dark chamber was found to be shorter than sham (*p* = .006) and L‐NAME (*p* = .002) groups, whereas the time spent in the dark chamber was significantly longer as compared to all the other groups (Figure 4b and c).

### 3.2 Open field test

**FIGURE 5:**
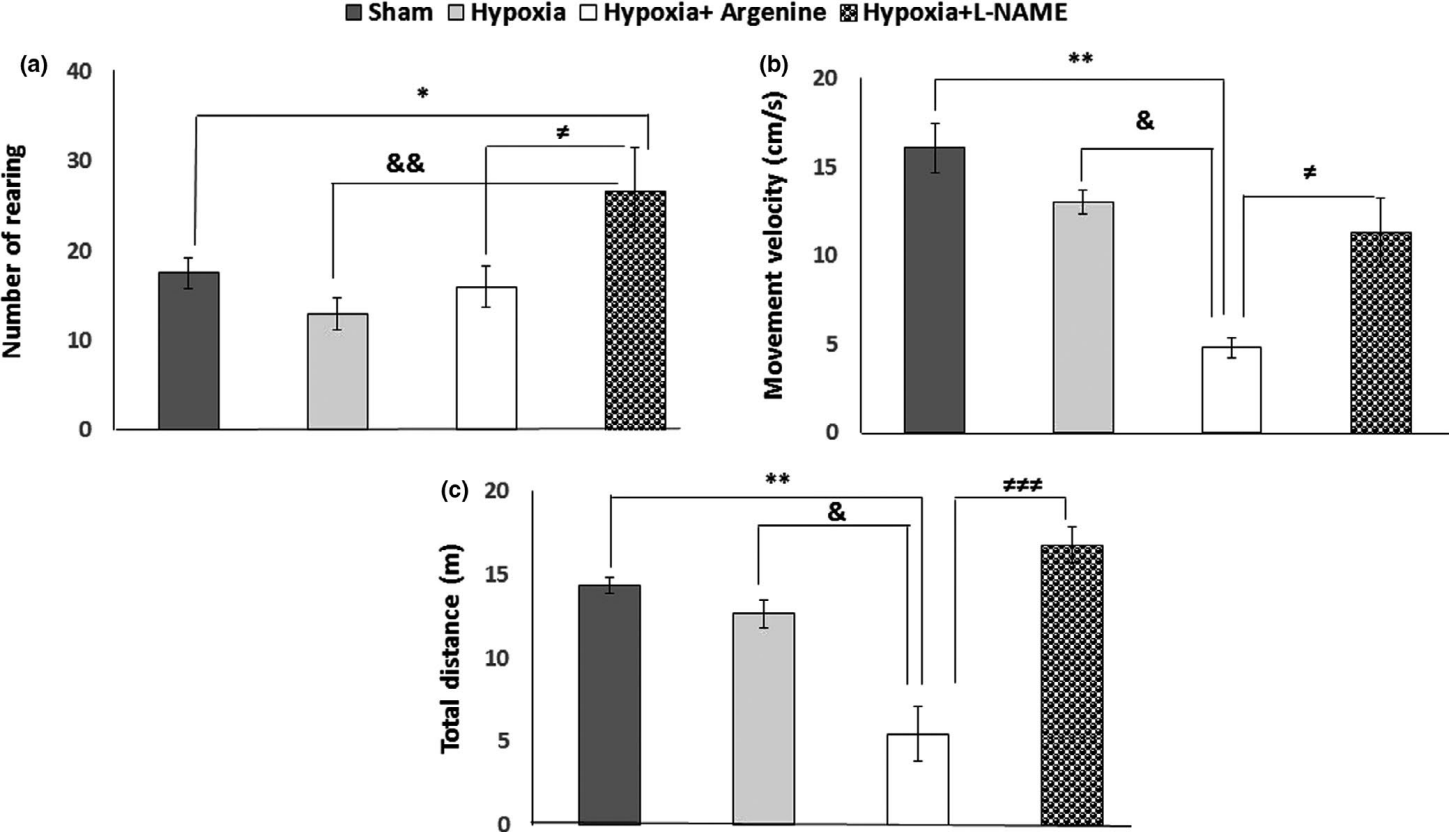
Locomotor activity in the open field test. (a) The number of rearing in the L‐NAME‐treated group was higher than the other groups. *p < .05 L‐NAME‐treated versus sham; &&*p* < .01 L‐NAME‐treated versus H and ≠*p *< .05 L‐NAME‐treated versus L‐arginine‐treated groups. (b) The average of movement velocity in the L‐arginine‐treated group was lower than the H and L‐NAME‐treated groups, *p* < .05 and the sham group, *p* < .01. (c) Total distance was decreased in the L‐arginine‐treated group compared to the sham (*p* < .01), hypoxia (*p* < .05), and L‐NAME‐treated (*p* < .001) groups

The result demonstrated that the number of rearing significantly increased in the L‐NAME‐treated group (26.71 ± 4.68) as compared to the sham group (17.42 ± 1.69; *p* = .04), hypoxia group (12.85 ± 1.78; *p* = .003), and L‐arginine‐treated group (15.87 ± 2.31; *p* = .012) (Figure 5a). L‐arginine injection in the hypoxia group had a major effect on the average of movement velocity (4.79 ± 0.55) as it was significantly lower than the sham (16.08 ± 1.38; *p* = .002), hypoxia (12.98 ± 2.68; *p* = .022), and L‐NAME‐treated (11.03 ± 3.98; *p* = .030) groups (Figure 5b). The total distance travelled in the open field box was also significantly decreased in the L‐arginine‐treated group as compared to the sham (14.39 ± 2.33; *p* = .002), hypoxia (12.65 ± 2.33; *p* = .012), and L‐NAME‐treated groups (16.77 ± 2.06; *p* = .000) (Figure 5c).

### 3.3 Rotarod test

**FIGURE 6:**
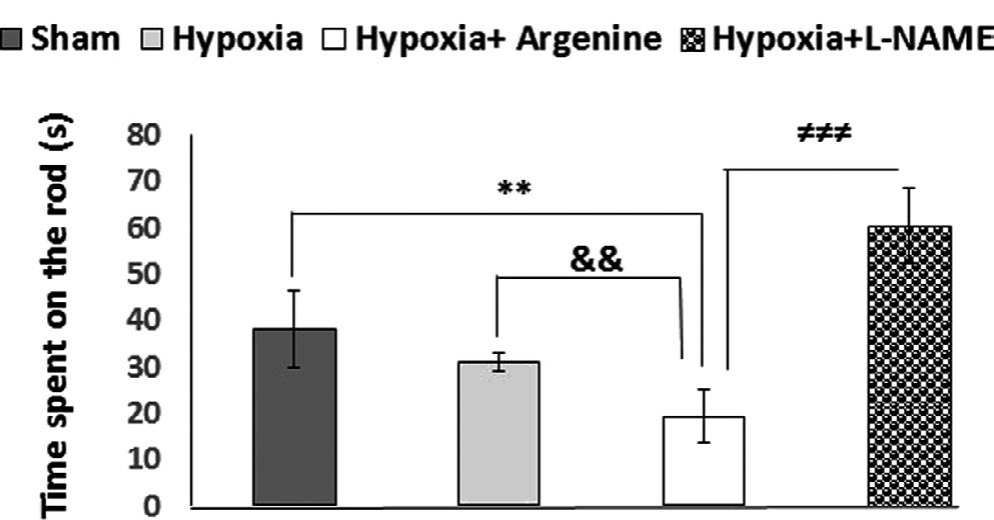
Motor performance deficits in the rotarod test in the L‐arginine‐treated group compared to the sham, hypoxia (*p* < .01), and L‐NAME‐treated (*p* < .001) groups

The rotarod test revealed that in comparison with the sham (38.08 ± 8.20; *p* = .004), hypoxia (31 ± 2.06; *p* = .002), and L‐arginine‐treated (19.31 ± 5.84; *p* = .000) groups, the animals that received L‐NAME antagonist (60.22 ± 8.12) spent more time on the rod get significantly increased in this group as compared to the other groups (Figure 6).

### 3.4 Histological analysis

**FIGURE 7:**
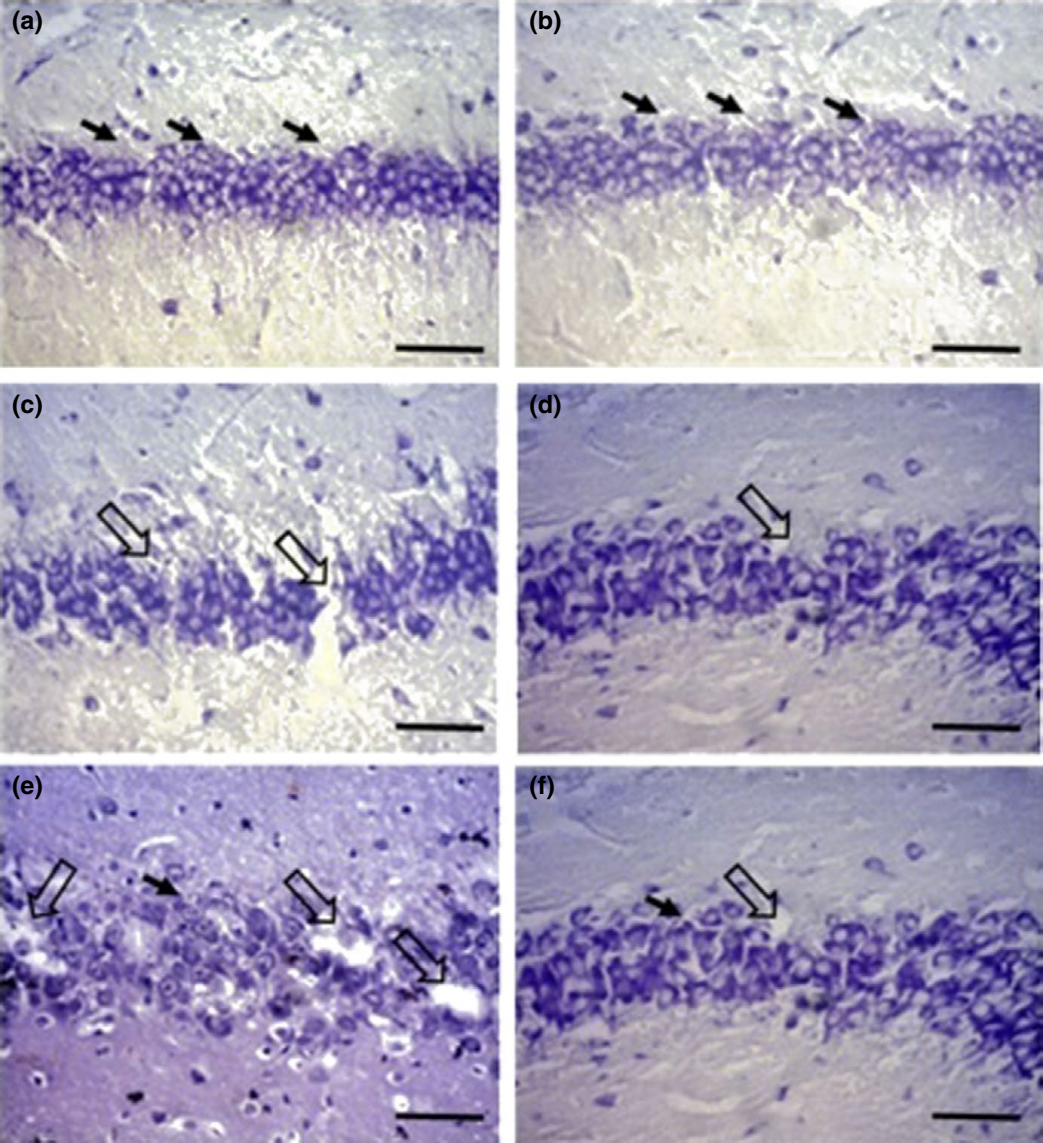
Nissl‐stained sections of the CA1 region in the hippocampus (200×). Normal histology of the CA1 was observed in the sham group at age 13 and 24 days (a and b). There was an extensive cell loss in the hypoxia group at P13 and P24 (c and d), and the number of cell death at P24 in the L‐arginine‐treated group was significantly higher (e). Antagonist injection in the hypoxia group (L‐NAME‐treated) showed higher cell number than the hypoxia and L‐arginine‐treated groups (f). Open arrows indicate the lack of cells, and black arrows show normal cells. Scale bar indicates 100 µm

**FIGURE 8:**
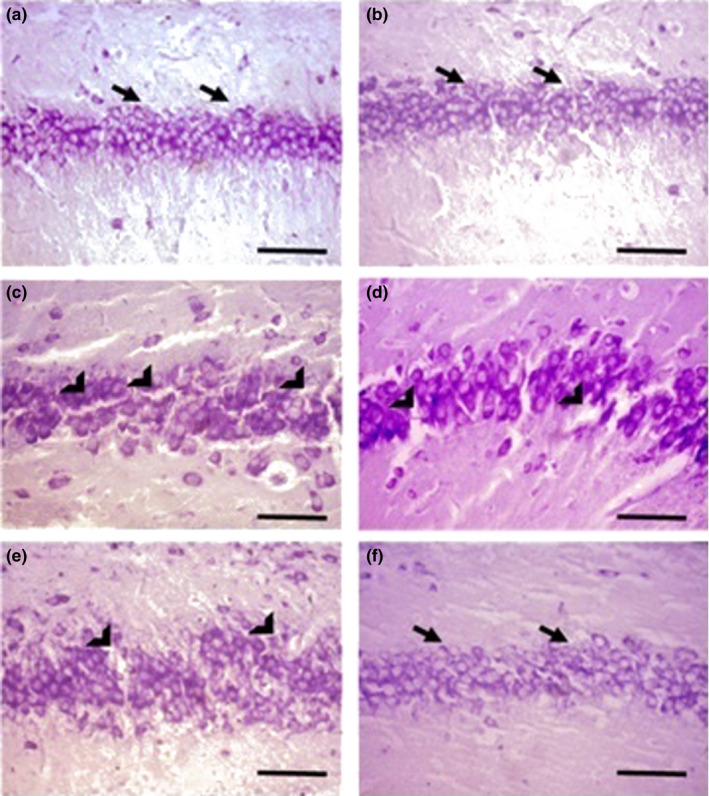
The CA1 H&E staining is shown in different groups at P13 and P24 (200×). (a) At P13 and P24, large and round cells were observed and there was no neuronal damage in the CA1 region of the rats in the sham group (a and b) (black arrows). In the hypoxia group, a significant neuronal loss was observed at both ages (c and d) (arrowhead) and the number of cells at P24 in the L‐arginine‐treated group (e) was significantly lower as compared to the L‐NAME‐treated group (f). Scale bar indicates 100 µm

The total number of pyramidal neurons per mm^2^ in the CA1 region was counted using Dinocapture imaging software (version 2.0) at a magnification of 200X. Both of the staining revealed that the number of pyramidal cells at P24 and P13 in the sham group was 280.4 ± 50.3 and 235.1 ± 15.7, respectively (Figures 7, 8a and b). The histology of the CA1 region was normal in this group, and no neuronal damage was observed. The average of pyramidal cells in the hypoxia group at P24 was 146.5 ± 24.0, showing a significant neural death as compared to the P13 (152.3 ± 12.5) (Figures 7, 8c and d). At P24, the number of cells (196.8 ± 23.7) was significantly higher in the L‐NAME‐treated group as compared to the hypoxia group (146.5 ± 24.0) and L‐arginine‐treated (76.5 ± 4.0) groups (Figures 7, 8e and f).

## 4 DISCUSSION

Neonatal hypoxia leads to the hippocampal damage in rats. Zhou et al. showed that hypoxia increased long‐term excitability in neurons. It has been well documented that neonatal hypoxia causes long‐term memory impairment by changing synaptic function and long‐term potentiation (LTP) in the neurons of the CA1 region of the hippocampus (Zhou et al., 2011). Our results demonstrated the similar findings as the hypoxia in neonatal rats precipitates the hippocampal damage which further leads to the memory and motor impairment. In contrast, we have observed that after NOS antagonist administration the behavioral impairment following hypoxia was attenuated.

The cognitive decline is one of the major outcomes of neonatal hypoxia. We have found that the hypoxia leads to the impairment in learning memory, and this is in line with previous studies as the Ikeda et al. also reported an increase in reference memory errors in the 8‐arm radial maze in rats exposed to ischemia‐hypoxia at the 7th postnatal day (Ikeda et al., 2000). Further, we had demonstrated that the administration of L‐arginine increased the susceptibility to the behavioral deficit as compared to the other groups. This confirms that the activation of NOS signaling can increase the intensity of hypoxia‐induced cognitive damage in neonatal rats. Similar results were observed with other behavioral test in open field test and rotarod test. This indicated that overstimulated NOS signaling can precipitates the chances to increase the functional abruption due to hypoxia.

NO signaling pathway plays a key role in regulating cerebral blood flow, and its impairment following ischemia‐hypoxia causes abnormality in physiological responses (Peers, Pearson, & Boyle, 2007). Energy depletion following hypoxia leads to oxidative stress and increases the production of free radicals and changes the functional performance of membrane pumps, these in turn increases the release of glutamate and leads to higher level of activation of NMDA receptors. As a result, the activity of inducible nitric oxide synthase (iNOS) increases in astrocytes and microglia. In addition, the production of cytokines and inflammatory factors such as TNFα and IFNβ enhances after hypoxia and induce further iNOS activity. As a result, the production of superoxide and peroxynitrite increases and damages proteins, lipids, and DNA of the neurons (Garry, Ezra, Rowland, Westbrook, & Pattinson, 2015).

Hypoxia results in progressive cellular and metabolic disorders including loss of neurons (especially in the hippocampus), altered synaptic function, increased excitability, the result of which is behavioral, sensory, motor, and cognitive impairments (Alwis, Johnstone, Yan, & Rajan, 2013). There are conflicting reports on the neuromodulatory role of NO in the behavioral responses, sensory‐motor system, and cognition. Some studies have reported that impaired activity of NOS is associated with neurodegenerative diseases such as Alzheimer's disease, and NOS inhibitors disrupt memory consolidation and LTP formation, whereas L‐arginine, as a NO precursor, improves memory formation (Asiimwe, Yeo, Kim, Jung, & Jeong, 2016). In 2008, Manukhina et al. investigated the role of NO on cognitive disorders induced by β‐amyloid injection. Their results suggested that administration of 20 mg/kg NOS inhibitor for 14 days (every other day) enhanced the destructive effects of β‐amyloid, whereas NOS agonist had a protective effect on memory impairment (Manukhina et al., 2008).

Our results are in agreement with studies that confirmed motor and memory impairment following hypoxia. Clinical evidence has also suggested long‐term sensory‐motor system deficit following hypoxia. In addition, a study reported that induction of hypoxia in rabbits at their late phase of pregnancy impaired movement and hypertonia in their kittens (Derrick et al., 2004). A large body of evidence suggests that synthesized NO by eNOS has a neuroprotective effect. However, both iNOS and nNOS are neurotoxic and can cause brain dysfunction (Garry et al., 2015). Under hypoxic conditions, nNOS activation is exacerbated and the excess of NO becomes neurotoxic. The probable mechanism for memory impairment following excess activation of NO synthesis is mediated by hyperactivation of glutamate receptors, which leads to excess Ca^2+^ entry, production of free radicals, mitochondrial dysfunction, and neural death (Dawson & Dawson, 1996). Histological results of this study demonstrated hippocampal damage following hypoxia. As previous studies reported, the hippocampus is one of the most important brain structures associated with memory formation and is highly sensitive to oxygen depletion. The hippocampus volume decreases after hypoxia, and pathological changes that occur immediately after hypoxia in the brain exacerbate memory dysfunction in childhood and later years of life span (Cooper et al., 2013). Different reports have demonstrated the association between the severity of hippocampal injury and memory impairment. Sever hypoxia has been shown to cause neuronal atrophy in the hippocampus, causing additional memory disruption (Isaacs et al., 2003). Another study reported that both the severity and duration of hypoxia were important factors in the resulted brain lesions. Short duration hypoxia only affected the cortex and hippocampus, and as the severity or duration of hypoxia increased, the lesion gradually extended to both brain hemispheres (Towfighi, Yager, Housman, & Vannucci, 1991).

In conclusion, the observed impairments in behavioral performance (memory and motor activity) and neuronal cell death in the hippocampus after neonatal hypoxic injury may be due to the fact that induction of hypoxia in a sensitive period of synaptic formation in the nervous system increases NO synthesis, evokes neurotoxicity pathways, especially those that cause mitochondrial dysfunction, and increases free radical production. The injection of the NOS agonist caused a higher level of brain tissue damage and disrupted behavioral responses through increased synthesis of NO. Finally, the NOS inhibitor showed possible therapeutic effects on the behavioral responses by preventing excess synthesis of NO and showing neuroprotective effects in important oxygen‐sensitive areas such as the hippocampus. Thus, our preliminary study suggests the critical involvement of NO in hypoxia‐induced structural alteration at hippocampus region and behavioral alteration in neonates.

## CONFLICT OF INTEREST

Authors declare that they have no conflict of interest.

## AUTHORS' CONTRIBUTIONS

Zohreh Ghotbeddin was responsible for study design, data analysis, evaluation, contributed behavioral experiments, approved the final manuscript, and responsible for overall supervision. Zahra Basir helped conduct the study and collection of histological data; data analysis and interpretation. Javad Jamshidian participated in study design, data collection, and conducted pharmacology experiments. Farideh Delfi contributed to experimental work and drafted the manuscript.

### PEER REVIEW

The peer review history for this article is available at https://publons.com/publon/10.1002/brb3.1841.

## Data Availability

The data that support the findings of this study are available from the corresponding author upon reasonable request.

## References

[brb31841-bib-0001] Alwis, D. S. , Johnstone, V. , Yan, E. , & Rajan, R. (2013). Diffuse traumatic brain injury and the sensory brain. Proceedings of the Australian Physiological Society, 44, 13–26. 10.1111/1440-1681.12100

[brb31841-bib-0002] Asiimwe, N. , Yeo, S. G. , Kim, M.‐S. , Jung, J. , & Jeong, N. Y. (2016). Nitric Oxide: Exploring the Contextual Link with Alzheimer’s Disease. Oxidative Medicine and Cellular Longevity, 2016, 1–10. 10.1155/2016/7205747 PMC5209623 28096943

[brb31841-bib-0003] Bale, T. L. , Contarino, A. , Smith, G. W. , Chan, R. , Gold, L. H. , Sawchenko, P. E. , … Lee, K.‐F. (2000). Mice deficient for corticotropin‐releasing hormone receptor‐2 display anxiety‐like behaviour and are hypersensitive to stress. Nature Genetics, 24, 410 10.1038/74263 10742108

[brb31841-bib-0004] Beckman, J. S. , Beckman, T. W. , Chen, J. , Marshall, P. A. , & Freeman, B. A. (1990). Apparent hydroxyl radical production by peroxynitrite: Implications for endothelial injury from nitric oxide and superoxide. Proceedings of the National Academy of Sciences, 87, 1620–1624. 10.1073/pnas.87.4.1620 PMC53527 2154753

[brb31841-bib-0005] Berg, A. T. (2011). Epilepsy, cognition, and behavior: The clinical picture. Epilepsia, 52, 7–12. 10.1111/j.1528-1167.2010.02905.x PMC3057769 21214534

[brb31841-bib-0006] Bolanos, J. P. , & Almeida, A. (1999). Roles of nitric oxide in brain hypoxia‐ischemia. Biochimica Et Biophysica Acta (BBA) ‐ Bioenergetics, 1411(2–3), 415–436. 10.1016/S0005-2728(99)00030-4 10320673

[brb31841-bib-0007] Bolaños, J. P. , Almeida, A. , Stewart, V. , Peuchen, S. , Land, J. M. , Clark, J. B. , & Heales, S. J. (1997). Nitric oxide‐mediated mitochondrial damage in the brain: Mechanisms and implications for neurodegenerative diseases. Journal of Neurochemistry, 68, 2227–2240. 10.1046/j.1471-4159.1997.68062227.x 9166714

[brb31841-bib-0008] Bon, C. L. , & Garthwaite, J. (2003). On the role of nitric oxide in hippocampal long‐term potentiation. Journal of Neuroscience, 23, 1941–1948. 10.1523/JNEUROSCI.23-05-01941.2003 12629199 PMC6741944

[brb31841-bib-0009] Butler, A. R. , & Williams, D. L. H. (1993). The physiological role of nitric oxide. Chemical Society Reviews, 22, 233–241. 10.1039/cs9932200233

[brb31841-bib-0010] Cooper, J. M. , Gadian, D. G. , Jentschke, S. , Goldman, A. , Munoz, M. , Pitts, G. , … Deanfield, J. (2013). Neonatal hypoxia, hippocampal atrophy, and memory impairment: Evidence of a causal sequence. Cerebral Cortex, 25, 1469–1476. 24343890 10.1093/cercor/bht332 PMC4428295

[brb31841-bib-0011] Dawson, V. L. , & Dawson, T. M. (1996). Nitric oxide neurotoxicity. Journal of Chemical Neuroanatomy, 10, 179–190. 10.1016/0891-0618(96)00148-2 8811421

[brb31841-bib-0012] Derrick, M. , Luo, N. L. , Bregman, J. C. , Jilling, T. , Ji, X. , Fisher, K. , … Back, S. A. (2004). Preterm fetal hypoxia‐ischemia causes hypertonia and motor deficits in the neonatal rabbit: A model for human cerebral palsy? Journal of Neuroscience, 24, 24–34. 10.1523/JNEUROSCI.2816-03.2004 14715934 PMC6729589

[brb31841-bib-0013] Fischer, A. H. , Jacobson, K. A. , Rose, J. , & Zeller, R. (2008). Hematoxylin and eosin staining of tissue and cell sections. Cold Spring Harbor Protocols, 2008(6), pdb.prot4986 10.1101/pdb.prot4986 21356829

[brb31841-bib-0014] Gale, S. D. , & Hopkins, R. O. (2004). Effects of hypoxia on the brain: Neuroimaging and neuropsychological findings following carbon monoxide poisoning and obstructive sleep apnea. Journal of the International Neuropsychological Society, 10, 60–71. 10.1017/S1355617704101082 14751008

[brb31841-bib-0015] Garry, P. , Ezra, M. , Rowland, M. , Westbrook, J. , & Pattinson, K. (2015). The role of the nitric oxide pathway in brain injury and its treatment—from bench to bedside. Experimental Neurology, 263, 235–243. 10.1016/j.expneurol.2014.10.017 25447937

[brb31841-bib-0016] Garthwaite, J. (1991). Glutamate, nitric oxide and cell‐cell signalling in the nervous system. Trends in Neurosciences, 14, 60–67. 10.1016/0166-2236(91)90022-M 1708538

[brb31841-bib-0017] Hosseini, M. , Headari, R. , Oryan, S. , Hadjzadeh, M. A. , Saffarzadeh, F. , & Khazaei, M. (2010). The effect of chronic administration of L‐arginine on the learning and memory of estradiol‐treated ovariectomized rats tested in the morris water maze. Clinics, 65, 803–807. 20835559 10.1590/S1807-59322020000800012 PMC2933129

[brb31841-bib-0018] Ikeda, T. , Xia, X. Y. , Xia, Y. X. , Ikenoue, T. , Han, B. , & Choi, B. H. (2000). Glial cell line‐derived neurotrophic factor protects against ischemia/hypoxia‐induced brain injury in neonatal rat. Acta Neuropathologica, 100, 161–167. 10.1007/s004019900162 10963363

[brb31841-bib-0019] Ikonomidou, C. , & Turski, L. (1996). Neurodegenerative disorders: Clues from glutamate and energy metabolism. Critical Reviews in Neurobiology, 10, 239–263. 8971131 10.1615/critrevneurobiol.v10.i2.50

[brb31841-bib-0020] Isaacs, E. , Vargha‐Khadem, F. , Watkins, K. , Lucas, A. , Mishkin, M. , & Gadian, D. (2003). Developmental amnesia and its relationship to degree of hippocampal atrophy. Proceedings of the National Academy of Sciences, 100, 13060–13063. 10.1073/pnas.1233825100 PMC240744 14555756

[brb31841-bib-0021] Kádár, A. , Wittmann, G. , Liposits, Z. , & Fekete, C. (2009). Improved method for combination of immunocytochemistry and Nissl staining. Journal of Neuroscience Methods, 184, 115–118. 10.1016/j.jneumeth.2009.07.010 19615409 PMC2753838

[brb31841-bib-0022] Kawase, M. , Kinouchi, H. , Kato, I. , Akabane, A. , Kondo, T. , Arai, S. , … Yoshimoto, T. (1996). Inducible nitric oxide synthase following hypoxia in rat cultured glial cells. Brain Research, 738, 319–322. 10.1016/S0006-8993(96)00924-9 8955528

[brb31841-bib-0023] Knowles, R. G. , & Moncada, S. (1994). Nitric oxide synthases in mammals. Biochemical Journal, 298, 249 10.1042/bj2980249 7510950 PMC1137932

[brb31841-bib-0024] Kumura, E. , Kosaka, H. , Shiga, T. , Yoshimine, T. , & Hayakawa, T. (1994). Elevation of plasma nitric oxide end products during focal cerebral ischemia and reperfusion in the rat. Journal of Cerebral Blood Flow & Metabolism, 14, 487–491. 10.1038/jcbfm.1994.60 8163591

[brb31841-bib-0025] Kuppusamy, P. , Ohnishi, S. T. , Numagami, Y. , Ohnishi, T. , & Zweier, J. L. (1995). Three‐Dimensional Imaging of Nitric Oxide Production in the Rat Brain Subjected to Ischemia—Hypoxia. Journal of Cerebral Blood Flow & Metabolism, 15, 899–903. 10.1038/jcbfm.1995.114 7593349

[brb31841-bib-0026] Manukhina, E. , Pshennikova, M. , Goryacheva, A. , Khomenko, I. , Mashina, S. Y. , Pokidyshev, D. , & Malyshev, I. Y. (2008). Role of nitric oxide in prevention of cognitive disorders in neurodegenerative brain injuries in rats. Bulletin of Experimental Biology and Medicine, 146, 391–395. 10.1007/s10517-009-0315-7 19489304

[brb31841-bib-0027] Odd, D. , Heep, A. , Luyt, K. , & Draycott, T. (2017). Hypoxic‐ischemic brain injury: Planned delivery before intrapartum events. Journal of Neonatal‐Perinatal Medicine, 10, 347–353. 10.3233/NPM-16152 29286930

[brb31841-bib-0028] Peers, C. , Pearson, H. A. , & Boyle, J. P. (2007). Hypoxia and Alzheimer’s disease. Essays in Biochemistry, 43, 153–164. 10.1042/bse0430153 17705799

[brb31841-bib-0029] Prast, H. , & Philippu, A. (2001). Nitric oxide as modulator of neuronal function. Progress in Neurobiology, 64, 51–68. 10.1016/S0301-0082(00)00044-7 11250062

[brb31841-bib-0030] Rustay, N. R. , Wahlsten, D. , & Crabbe, J. C. (2003). Influence of task parameters on rotarod performance and sensitivity to ethanol in mice. Behavioural Brain Research, 141, 237–249. 12742261 10.1016/s0166-4328(02)00376-5

[brb31841-bib-0031] Sedláčková, N. , Krajčiová, M. , Koprdová, R. , Ujházy, E. , Brucknerová, I. , & Mach, M. (2014). Subchronic perinatal asphyxia increased anxiety‐and depression‐like behaviors in the rat offspring. Neuroendocrinology Letters, 35, 2. 25638390

[brb31841-bib-0032] Shetty, J. (2015). Neonatal seizures in hypoxic–ischaemic encephalopathy–risks and benefits of anticonvulsant therapy. Developmental Medicine & Child Neurology, 57, 40–43. 10.1111/dmcn.12724 25800491

[brb31841-bib-0033] Sohrabi, A. J. , Ghotbeddin, Z. , & Tabandeh, M. R. (2017). Study the effect of crocin on avoidance memory and motor activity impairment induced by doxorubicin administration in adult male rats. Arak Medical University Journal, 20, 45–56.

[brb31841-bib-0034] Towfighi, J. , Yager, J. , Housman, C. , & Vannucci, R. (1991). Neuropathology of remote hypoxic‐ischemic damage in the immature rat. Acta Neuropathologica, 81, 578–587. 10.1007/BF00310141 1858486

[brb31841-bib-0036] Yao, C. , Shi, X. , Zhang, Z. , Zhou, S. , Qian, T. , Wang, Y. , … Yu, B. (2016). Hypoxia‐induced upregulation of miR‐132 promotes Schwann cell migration after sciatic nerve injury by targeting PRKAG3. Molecular neurobiology, 53(8), 5129–5139. 26399639 10.1007/s12035-015-9449-y

[brb31841-bib-0035] Zhou, C. , Bell, J. J. L. , Sun, H. , & Jensen, F. E. (2011). Hypoxia‐induced neonatal seizures diminish silent synapses and long‐term potentiation in hippocampal CA1 neurons. Journal of Neuroscience, 31, 18211–18222. 10.1523/JNEUROSCI.4838-11.2011 22171027 PMC3282023

